# Neuroprotective Potency of Neolignans in *Magnolia officinalis* Cortex Against Brain Disorders

**DOI:** 10.3389/fphar.2022.857449

**Published:** 2022-06-16

**Authors:** Shun Zhu, Fang Liu, Ruiyuan Zhang, Zongxiang Xiong, Qian Zhang, Li Hao, Shiyin Chen

**Affiliations:** ^1^ State Key Laboratory of Southwestern Chinese Medicine Resources, College of Pharmacy, Chengdu University of Traditional Chinese Medicine, Chengdu, China; ^2^ Huarun Sanjiu (ya’an) Pharmaceutical Group Co., LTD., Ya’an, China; ^3^ Department of Orthopedics of Traditional Chinese Medicine, Sichuan Provincial People’s Hospital, University of Electronic Science and Technology of China, Chengdu, China

**Keywords:** neolignans, *Magnolia officinalis*, neuroprotective effect, multiple pathways, brain disorders

## Abstract

In recent years, neurological diseases including Alzheimer’s disease, Parkinson’s disease and stroke are one of the main causes of death in the world. At the same time, the incidence of psychiatric disorders including depression and anxiety has been increasing. Accumulating elderly and stressed people suffer from these brain disorders, which is undoubtedly a huge burden on the modern aging society. Neolignans, the main active ingredients in *Magnolia officinalis* cortex, were reported to have neuroprotective effects. In addition, the key bioactive ingredients of neolignans, magnolol **(1)** and honokiol **(2)**, were proved to prevent and treat neurological diseases and psychiatric disorders by protecting nerve cells and brain microvascular endothelial cells (BMECs). Furthermore, neolignans played a role in protecting nerve cells *via* regulation of neuronal function, suppression of neurotoxicity, etc. This review summarizes the neuroprotective effect, primary mechanisms of the leading neolignans and provides new prospects for the treatment of brain disorders in the future.

## Graphical Abstract

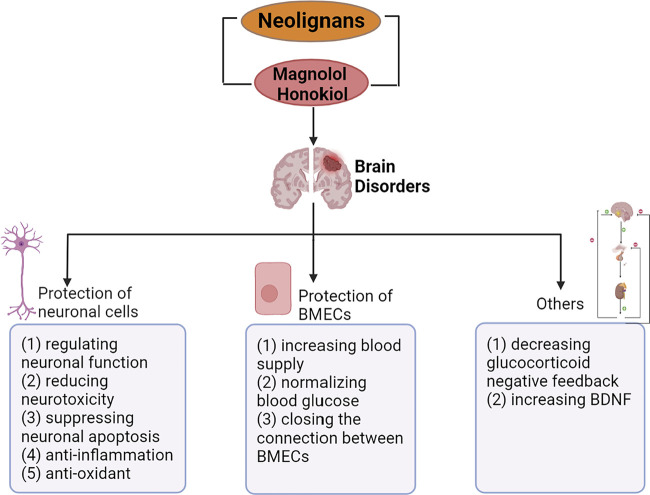

## 1 Introduction

Brain disorders including Alzheimer’s disease (AD), Parkinson’s disease (PD), stroke, anxiety, depression, are the diseases with increasing incidence in recent years. AD is one of the most common forms of dementia in the aging population, cause a growing death toll (Walsh and Selkoe, 2004; Oboudiyat et al., 2013; Silva et al., 2019; World Health Organization, 2020). From 1990 to 2015, the number of patients with PD approximately doubled due to the aging population (Dorsey et al., 2018). Cerebral vascular occlusion and rupture are the causes of stroke, followed by a series of nerve injuries. According to WHO, stroke alone accounted for 11% of the world’s total deaths in 2019 (World Health Organization, 2020). Anxiety and depression caused by great pressure are major psychiatric disorders, affecting 21% of the population in some developed countries annually (Alexeev et al., 2012; Cheng et al., 2018). The main therapeutic drugs for AD were cholinesterase inhibitors, memantine and so on (Taipale et al., 2016). Long term use of certain drugs in patients with AD might lead to drug interaction and toxicity (Cacabelos et al., 2008). Levodopa or other dopamine agonists were usually used as dopamine substitutes to treat PD, which often resulted in dopaminergic adverse reactions including involuntary movements, response fluctuations, etc. (Clarke 2004) When stroke occurred, it was usually treated by reperfusion and surgery, while there were few studies on chronic phase treatment (Kuriakose and Xiao, 2020; Szelenberger et al., 2020). Anti-anxiety drugs including benzodiazepines and anti-depressants including selective serotonin reuptake inhibitors were widely used in the pharmaceutical market (Nemeroff 2007; Ku et al., 2011). Sedation, cognitive and memory impairment, and other side effects often occurred after long-term medication (Lader 2008; Li L. F. et al., 2013; Millan et al., 2015). Therefore, the importance of constantly searching for new effective components for the treatment of brain disorders should be realized.

Neolignans are principal active ingredients of *Magnolia officinalis* cortex. Magnolol (MN) **(1)** and honokiol (HK) **(2)** are the most widely studied bioactive substances in neolignans of *Magnolia officinalis*. Current researches demonstrated that pharmacological activities of MN **(1)**, HK **(2)**-based neolignans included gastrointestinal protection, anti-bacterial, anti-inflammatory, anti-oxidation, cardiovascular protection and anti-tumor (Luo et al., 2019; Rauf et al., 2021). *Magnolia officinalis* extract mainly including neolignans was used as an overall wellness nutritional supplement in United States and the recommended dosage was 200–800 mg/day per person for a variety of maladies including anxiety, depression, diabetes, inflammation, headache, muscle pain, weight loss, asthma, stroke, bacterial infection etc. (Poivre and Duez, 2017; Bui et al., 2020) In addition, neolignans were often available for daily life and added to mints, gums, mouthwash, insecticide, cosmetic products (Ye et al., 2015; Poivre and Duez, 2017; Bui et al., 2020; Ghorbani et al., 2021). Recently, accumulating studies showed that neolignans manifest neuroprotection on some brain disorders.

Neolignans have neuroprotective effects on brain disorders including AD, PD, stroke, anxiety, depression. AD is often accompanied by abnormal cholinergic nerve function, abnormal production and deposition of beta-amyloid (Aβ) and neuroinflammation. PD is characterized by the formation of Lewy bodies and dopaminergic neurological dysfunction (Xian et al., 2015). Stroke leads to neuroinflammation, oxidative stress, apoptosis and damages to BMECs, which can further damage the nerve (Xiaoyan 2016; Kou et al., 2017; Huang et al., 2018). Anxiety is mainly caused by abnormal GABAergic nerve function, and the improvement of serotonergic nerve function can play a therapeutic role in depression (Alexeev et al., 2012; Bai et al., 2018). It is concluded that the therapeutic effects of neolignans on these diseases can be divided into effects on nerve cells and brain microvascular endothelial cells (BMECs). The effects of neolignans on nerve cells include regulation of various neuronal function, reduction of neurotoxicity of Aβ and other protein deposition, suppression of apoptosis, neuroinflammation and nerve oxidation. While, the effects of neolignans on BMECs include normalization of blood glucose levels, promotion of vasodilation and reinforcement of tight junctions between BMECs. From the perspective of the mechanism of treating these diseases, this review mainly summarizes the neuroprotective effects of neolignans at the cellular level including nerve cells and BMECs.

## 2 Literature and Data Search Methodology

The comprehensive information of this review came from electronic databases including the Web of Science (http://wokinfo.com/), ScienceDirect (www.sciencedirect.com), PubMed (http://ncbi.nlm.nih.gov/), CNKI (http://cnki.net/), Google Scholar (http://scholar.google.com.cn/) by using keywords “*Magnolia officinalis*,” “Neolignans,” “Houpo,” “Magnolol,” “Honokiol,” “Neurological disease,” “Psychiatric disorders,” “Alzheimer,” “Parkinson,” “Stroke,” “Anxiety,” “Depression” and their combinations.

## 3 Chemistry of Principal Bioactive Ingredients in Neolignans

**FIGURE 1:**
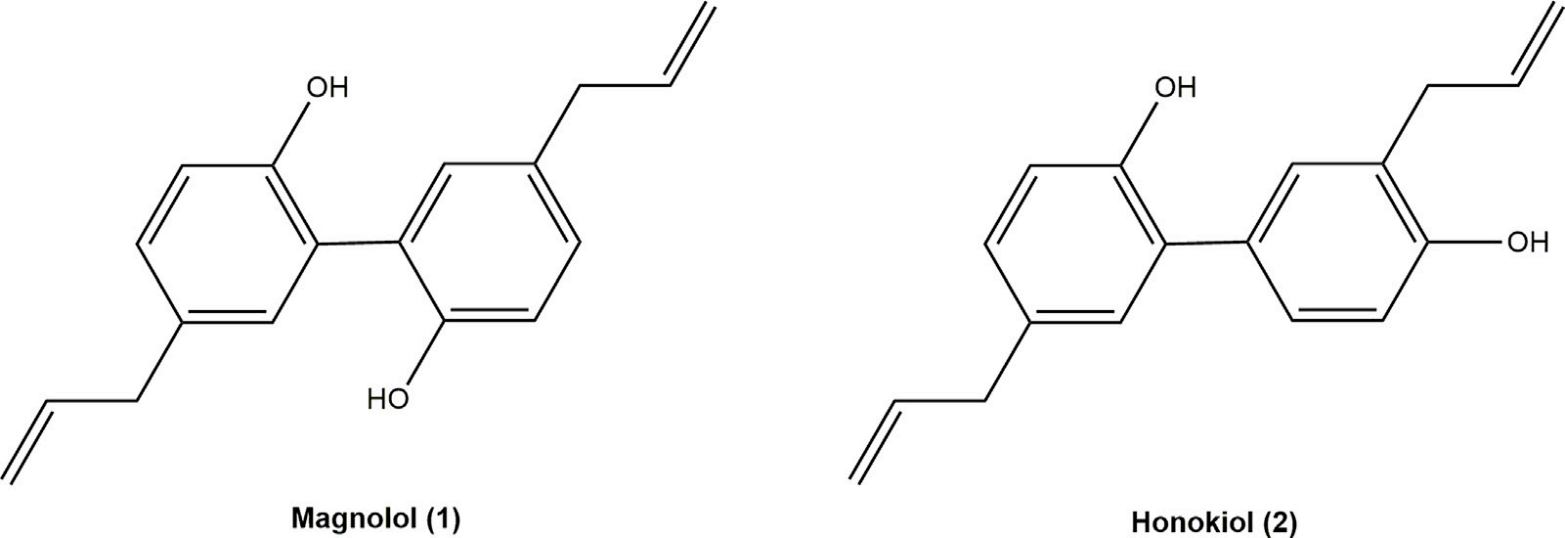
Chemical structures of magnolol and honokiol.

Neolignans are polymerized by the side chain of one phenylpropylene and the benzene ring of another phenylpropylene. MN **(1)** (5,5′-Diallyl-2,2′-dihydroxybiphenyl) and HK **(2)** (5,3′-Diallyl-2,4′-dihydroxybiphenyl), two polyphenol compounds, are the leading bioactive ingredients of neolignans (Figure 1). Neolignans are a large class of natural compounds derived from the oxidative coupling of two C6–C3 units (Teponno et al., 2016). Their unique pharmacophore structure is two phenolic rings linked through a C–C bond, allowing the interaction with various biological targets (Hajduk et al., 2000). MN **(1)** and HK **(2)** were proved to have a series of pharmacological effects, including anti-cancer, anti-oxidant, anti-inflammatory, neuroprotective (Lin et al., 2007; Shen et al., 2010; Amorati et al., 2015; Ranaware et al., 2018; Cardullo et al., 2020). Moreover, MN **(1)** and HK **(2)** are isomers, whose only difference is the relative position of one hydroxyl group and allyl group. The 4′-hydroxy group and the 5-allyl group are the key to higher neurotrophic activity of HK **(2)** than that of MN **(1)** (Fukuyama et al., 2020). Furthermore, both HK **(2)** and MN **(1)** exerted neuroprotective effects, which might be related to penetrate the blood-brain barrier (BBB) (Kantham et al., 2017).

## 4 Mechanism Underlying the Effects of Neolignans in NDs

The major defining neuropathological features of AD are beta-amyloid (Aβ) accumulation in the cerebral cortex and hippocampus. In addition, accumulating researches showed that cholinergic injury and dysfunction of hippocampus neurons were the critical causes of weakened function of learning and memory (Mohapel et al., 2005; Gökçek-Saraç et al., 2012). Studies showed that neolignans could protect neurons of AD patients by reducing Aβ toxicity, regulating cholinergic nerve function, anti-inflammatory, anti-oxidant, etc. Lewy bodies involving the aggregation of α-synuclein (αS) into amyloid structures are a pathological hallmark of PD (Volpicelli-Daley et al., 2011; Luk et al., 2012; Iyer et al., 2014). Neolignans can regulate dopamine neurons and reduce αS toxicity to protect neurons. Neolignans treat stroke by protecting brain microvascular endothelial cells (BMECs) and inhibiting inflammation and oxidative stress caused by stroke. It is proved that neolignans can modulate HPA axis and regulate GABAergic nerves, treating anxiety and depression. In general, neolignans prevent and treat brain disorders mainly by protecting nerve cells and BMECs.

### 4.1 Protection of Neuronal Cells

Neolignans protect nerve cells mainly through regulation of neuronal function, reduction of neurotoxicity, suppression of apoptosis, anti-inflammatory and anti-oxidation. Neolignans flexibly regulate acetylcholine and cholinesterase activity to protect cholinergic neurons; prevent striatal degeneration to protect dopamine neurons; enhance GABA by modulating the benzodiazepine site of GABA receptors and directly increasing GABA neurotransmission; improve serotonergic neurotransmission by increasing levels of 5-HT, 5-HT receptors, and brain-derived neurotrophic factor. Besides, neolignans decline Aβ toxicity through reducing APP, γ-secretase, BACE1 and increasing lysosomal degradation; reduce αS toxicity through stabilizing αS native conformations, leading to the reduction of neurotoxicity. In addition, neolignans reduce the levels of Bcl-2-associated X protein, caspase-3 and other apoptosis-related proteins, thereby inhibiting apoptosis. Moreover, neolignans achieve anti-inflammatory effect by down-regulating nuclear factor-κB and anti-oxidant by reducing reactive oxygen species.

#### 4.1.1 Regulation of Neuronal Function

**FIGURE 2:**
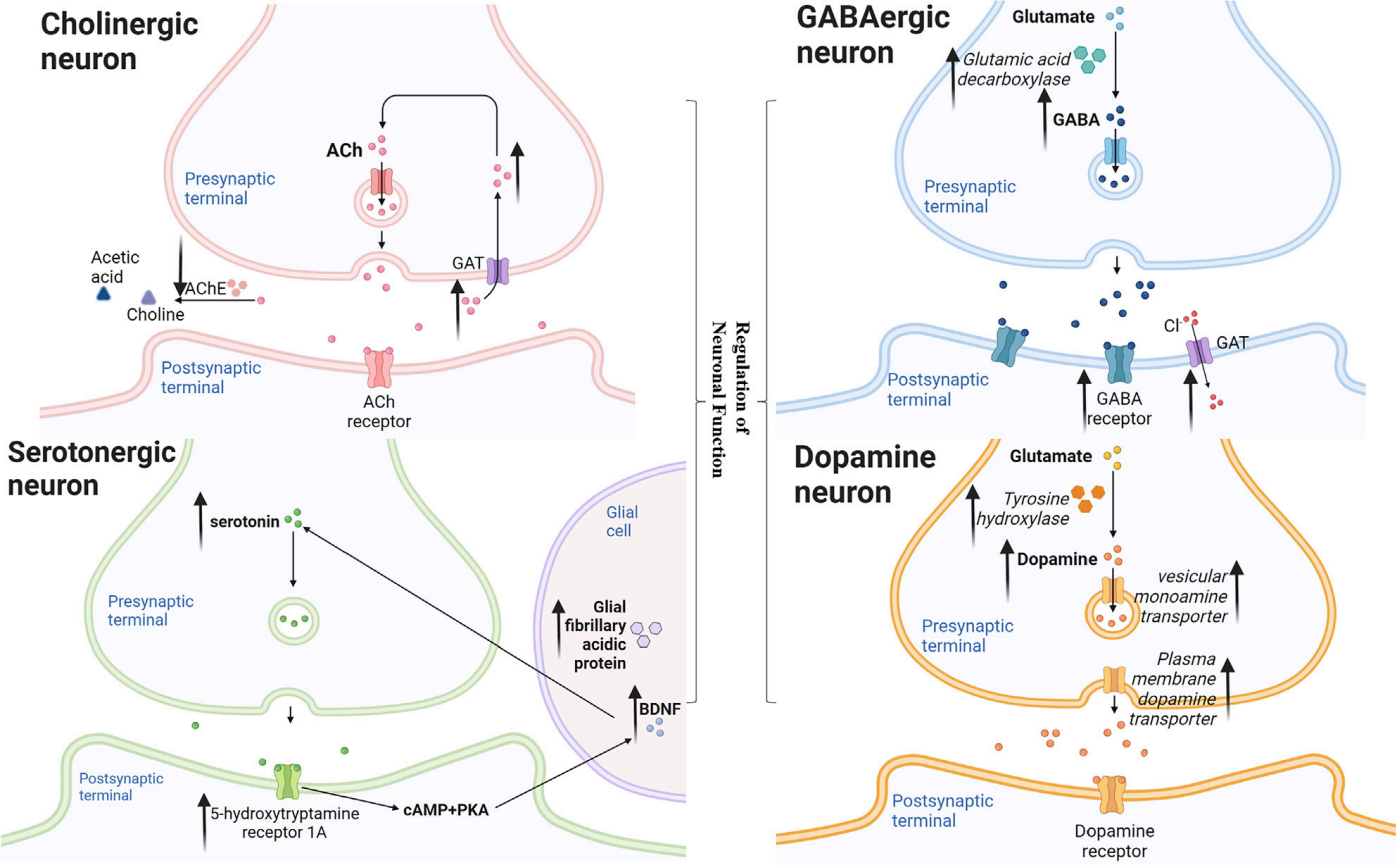
Mechanisms involved in the regulation of neuronal function by neolignans.

The main pathway diagram is shown in Figure 2.

##### 4.1.1.1 Cholinergic Neuron

It has been established that the disadvantage in cholinergic system was likely the most general pathogenesis of AD (Walsh and Selkoe, 2004). But the cholinergic activity was even upregulated in the brain at the earliest stages of AD (Frölich 2002). In the passive avoidance and the Morris water maze tests, MN **(1)** maintained acetyl cholinesterase (AChE) positive nerve fiber density and AChE activity in hippocampus homogenate, thereby reversing memory impairment induced by scopolamine (Scop) in mice (Li Y. S. et al., 2013). It suggested that early therapeutic effect of MN **(1)** on AD was more suitable than AChE antagonist alone. On another stage, HK **(2)** could inhibit the activity of AChE and increased the level of acetylcholine (ACh) in the brain tissues of the Scop-treated mice (Xian et al., 2015). In another study, MN **(1)** or HK **(2)** were orally administered to SAMP8 mice once a day for 14 days, then researchers got consistent result. Besides, MN **(1)** and HK **(2)** compensated for the neurotrophic function of septal-hippocampal pathway and inhibited apoptosis by activating neurotrophin-mediated Akt pathway. (Matsui et al., 2009). These findings suggested that MN **(1)** and HK **(2)** maight be used cooperatively in the different states of AD.

##### 4.1.1.2 Dopamine Neuron

The selective loss of nigral dopaminergic neurons resulted in a decrease of striatal dopamine, which could lead to PD (Lansbury and Brice, 2002). The plasma membrane dopamine transporter (DAT), tyrosine hydroxylase (TH) and the vesicular monoamine transporter (VMAT2) involved in the release, synthesis and removal of dopamine were essential for normal dopamine neurotransmission. Thus, the selective degeneration of DAT-, TH- and VMAT2-expressing in dopamine nerve terminals was thought to underlie PD (Miller et al., 1999; Chen et al., 2018). MN **(1)** was reported to attenuate the decrease in DAT and TH protein levels in the striatum, repressing MPTP-induced and 6-OHDA-lesioned neurodegeneration in mice and MPP+-induced cytotoxicity to human neuroblastoma SH-SY5Y cells (Chen et al., 2011; Muroyama et al., 2012). HK **(2)** ameliorated motor dysfunction in 6−OHDA-lesion hemiparkinsonian mice with higher DAT, TH and VMAT2 levels. Besides, HK **(2)** attenuated the increase of glial fibrillary acidic protein (GFAP) which is the marker of reactive astrocytes and nerve injury. In addition, these effects were found to impede by PPAR-γ antagonist (Chen et al., 2018). It suggested that MN **(1)** and HK **(2)** might partly and HK reverse dopaminergic nerve damage at least partly through PPAR-γ pathway.

##### 4.1.1.3 GABAergic Neuron

It has been established that γ-aminobutyric acid (GABA), an important inhibitory neurotransmitter, could act on GABA_A_ receptor to open Cl^−^-channel, thereby hyperpolarizing neurons and playing the role of sedation and anti-anxiety. Researchers found that MN **(1)** and HK **(2)** enhanced both phasic and tonic GABAergic neurotransmission in hippocampal dentate granule neurons of rats, which could be abolished by an antagonist at the benzodiazepine site of the GABA_A_ receptor (Alexeev et al., 2012; Qu et al., 2012). Furthermore, a HK **(2)** derivative was reported to increased ^36^Cl^−^ influx in mouse cerebral cortical synaptoneurosome (Maruyama et al., 2001). Interestingly, Ku et al. (2011) found that the anti-anxiety effect of HK **(2)** was similar to diazepam by the treatment of mice injected 7 days. Besides, the activity of hippocampal GABA synthesized enzymes of HK **(2)** treated mice was significantly increased than that of diazepam treated groups. In general, neolignans enhance GABAergic neurotransmission by modulating the benzodiazepine site of the GABA_A_ receptor as well as directly increasing GABA.

##### 4.1.1.4 Serotonergic Neuron

Glial cells could produce brain-derived neurotrophic factor (BDNF), which had principle neurotrophic and supportive effects on neurons (Althaus and Richter-Landsberg, 2000). It was reported that in unpredictable chronic mild stress rats, MN **(1)** increased the level of glial fibrillary acidic protein (GFAP) that was an important marker and component of glial cells (Li L. F. et al., 2013). Accumulating studies have shown that the improvement of the serotonin (5-HT) system was regarded as one of the therapeutic targets for depression (Li L. F. et al., 2012). Serotonergic fibers densely dominated the hippocampus, which played an important role in emotional and cognitive processes (Xia et al., 2019). Abundant evidence suggested that the decrease of 5-HT neurotransmission or receptor expression could lead to depression (Dale et al., 2016). MN **(1)** and HK **(2)** were reported to effectively improve the levels of 5-HT. In a study, administration of MN **(1)** normalized the serotonergic system changes in the unpredictable chronic mild stress-treated rats. And in another research, HK **(2)** was found to have the similar effect. (Xu et al., 2008; Bai et al., 2018). 5-hydroxytryptamine receptor 1A (HTR1A) is a 5-HT receptor which has the highest affinity for 5-HT among all 5-HT receptor subtypes (Wang et al., 2016). Xia et al. (2019) found that a Chinese medicine containing MN **(1)** increased HTR1A protein and mRNA expression. cAMP-response element binding (CREB) is a key regulator of neuronal growth and related to the expression of BDNF. HTR1A could regulate cyclic adenosine monophosphate (cAMP)/protein kinase A (PKA)/CREB pathway in hippocampus. cAMP activated PKA and PKC subunits into the nucleus, phosphorylated and activated CREB transcription factor-specific serine residues to bind to the cAMP response element and then improved BDNF expression (Wang C. et al., 2017). On the other hand, BDNF had potent neurotrophic effects on serotonergic nerve and promoted the growth and differentiation of nerve synapses (Huang and Reichardt, 2001).

#### 4.1.2 Reduction of Neurotoxicity

**FIGURE 3:**
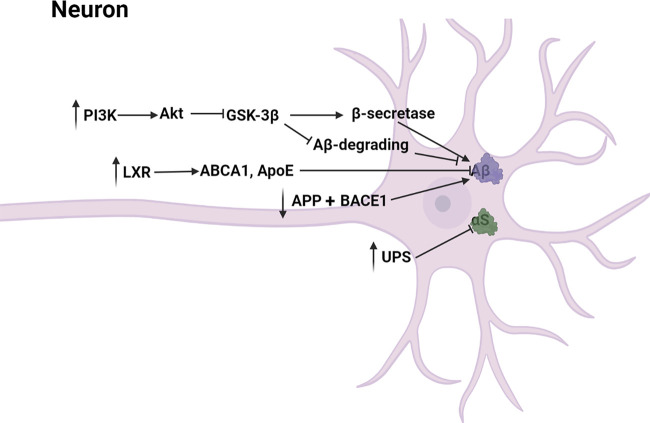
Mechanisms involved in the reduction of neurotoxicity by neolignans.

The main pathway diagram is shown in Figure 3.

##### 4.1.2.1 Aβ Toxicity

Abnormal production and deposition of beta-amyloid (Aβ) induced neurotoxicity, leading to the irreversible degeneration and apoptosis of neurons (Tanokashira et al., 2017). Specifically, Aβ induced microglial and nuclear factor-κB (NF-κB) activation which contributed to the increase of inflammatory mediators including COX-2, ROS, iNOS, NO and prostaglandin E2 (PGE-2) (Maezawa et al., 2011; Cai et al., 2014). Besides, Aβ induced production of hydrogen peroxide from NADPH oxidase (Jekabsone et al., 2006). MN **(1)** has been proved to dose-dependently reduce Aβ deposition, toxicity and memory impairment caused by Aβ in transgenic *C. elegans*. In another experiment, HK **(2)** was found to have similar potency to MN **(1)** by protecting against Aβ-induced toxicity in transgenic *C. elegans* (Gökçek-Saraç et al., 2012; Kantham et al., 2017). Besides, ethanol extract of *Magnolia officinalis* including MN **(1)**, HK **(2)** and other derivatives prevented memory dysfunction and amyloidogenesis in AD mouse model (Lee et al., 2013; Kubo 2015). Aβ was generated through successive cleavages of amyloid precursor protein (APP) by β-site APP cleaving enzyme 1 (BACE1) and γ-secretase (De Strooper et al., 2010). It has been reported that MN **(1)** and HK **(2)** might downregulate APP and BACE1 to block the generation of Aβ. (Lawrence 2009; Oboudiyat et al., 2013; Wang M. et al., 2017; Xie et al., 2020). Moreover, MN **(1)** inhibited the activities of β-secretase and γ-secretase and enhanced the Aβ-degrading enzymatic activities via adjusting the PI3K/Akt/GSK-3β pathway (Aggarwal et al., 2012; Xian et al., 2020). LXR, the target gene of PPAR-γ, was activated by MN **(1)** to upregulate other genes including ABCA1 and ApoE which mediated the lysosomal clearance of Aβ, reducing Aβ toxicity (Cramer et al., 2012; Oboudiyat et al., 2013; Bonet-Costa et al., 2016; Zhang et al., 2018; Xie et al., 2020). In general, MN **(1)** and HK **(2)** declined Aβ toxicity through reducing APP-, γ-secretase- and BACE1-mediated production of Aβ and increasing lysosomal-mediated degradation of Aβ.

##### 4.1.2.2 αS Toxicity

Lewy bodies were the pathological marker of PD and the most abundant protein was α-Synuclein (αS) (Ganguly et al., 2021). Excessive αS disrupted the interactions of mitochondria and ER, leading to the decrease of mitochondrial ATP production and nerve cell injury (Paillusson et al., 2017). Ubiquitin-proteasome system (UPS), responsible for clearing mutated and misfolded proteins, was an important pathway involving protein degradation *in vivo*. It is reported that functional impairment of the UPS could lead to intracellular accumulation of α-synuclein probably by inhibiting its clearance in the proteasomal pathway (Ganguly et al., 2020). Over-expression of αS and functional impairment of the UPS in nerve cells reciprocally induced, then poisoned dopaminergic neurons, leading to PD (Bukhatwa et al., 2010; Egeler et al., 2011; Heo and Rutter, 2011; Taylor and Rutter, 2011). Rats were administered orally with Anchanling that was a Chinese medicine mainly containing MN **(1)**, then Li Y. et al. (2012) found that UPS function was increased and αS expression was decreased. *In vitro*, HK **(2)** was reported to inhibit αS amyloidogenic aggregation by stabilizing αS native conformations (Das et al., 2018). The specific mechanism of MN **(1)** and HK **(2)** inhibiting over-expression of αS still need to be further studied.

#### 4.1.3 Suppression of Apoptosis

**FIGURE 4:**
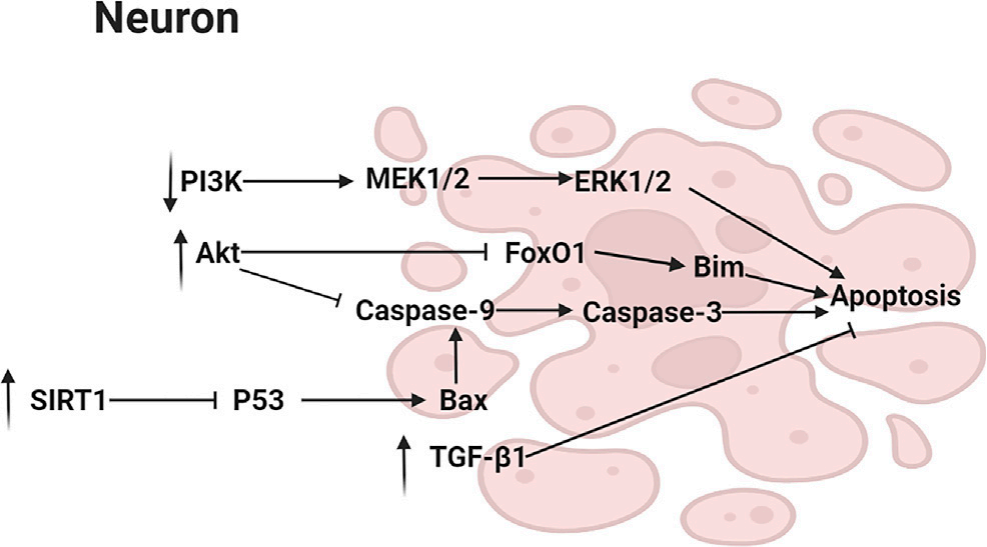
Mechanisms involved in the suppression of apoptosis by neolignans.

Caspase family belonged to cysteine protease which played an important role in Aβ-induced apoptosis *in vitro*. MN **(1)** and HK **(2)** were reported to inhibit the elevation of caspase-3 activity, inhibiting apoptosis (Hoi et al., 2010). Furthermore, it has been shown that one of MN **(1)** derivative inhibited apoptosis via reducing the activity of caspase-3 in ischemic cerebral tissue (Li et al., 2015). In another study, a HK **(2)** derivative was found to have similar anti-apoptosis effect (Bu et al., 2014). In cerebral ischemic stroke rat model, the protein expression of Bcl-2-associated X protein (Bax), an apoptosis-related factor, were reduced by MN **(1)** (Liu et al., 2018). Bax activated Caspase-9, which initiated a cascade that ultimately activated caspase-3. The activated executor, Caspase-3, led to apoptosis by hydrolysis of caspase target proteins. Furthermore, the anti-apoptotic effect of MN **(1)** was related to Silent information regulator 1 (SIRT1) activation, a histone deacetylase, which was related to several cellular physiological regulatory pathways (Kou et al., 2017). The activation of SIRT1 led to the reduction in p53, resulting in a decrease of Bax (Yang et al., 2021). In addition, SH-SY5Y human neuroblastoma cells were preincubated with various concentrations of MN **(1)** (8, 16, and 32 μM) for 2 h, then researchers found that MN **(1)** increased the phosphorylation of Akt and FOXO1, resulting in repressing FOXO-mediated growth arrest and apoptosis in neuronal cells. Blockage of PI3K could block the activation of Akt by MN **(1)**. It was also found that MN treatment suppressed ERK activation that was a pro-apoptosis signal mediator. These suggested that MN **(1)** protected models from apoptosis through activating the PI3K/Akt/FOXO1 pathway and inhibiting the MAPK/ERK pathway. Akt activated by PI3K phosphorylated and inhibited FOXO1, preventing FOXO1 activation from transactivating Bcl-2 family member Bim which produced apoptosis. Similarly, after treating SAMP8 mice with MN **(1)** or HK **(2)**, phosphorylation of Akt in the forebrain was enhanced (Matsui et al., 2009; Dong et al., 2013). Moreover, transforming growth factor β1 (TGF-β1), an anti-apoptosis factor, was increased by MN **(1)** in the ischemic cortex (Wang et al., 2013). However, its specific mechanism need be further studied. The main pathway diagram is shown in Figure 4.

#### 4.1.4 Anti-Neuroinflammation

**FIGURE 5:**
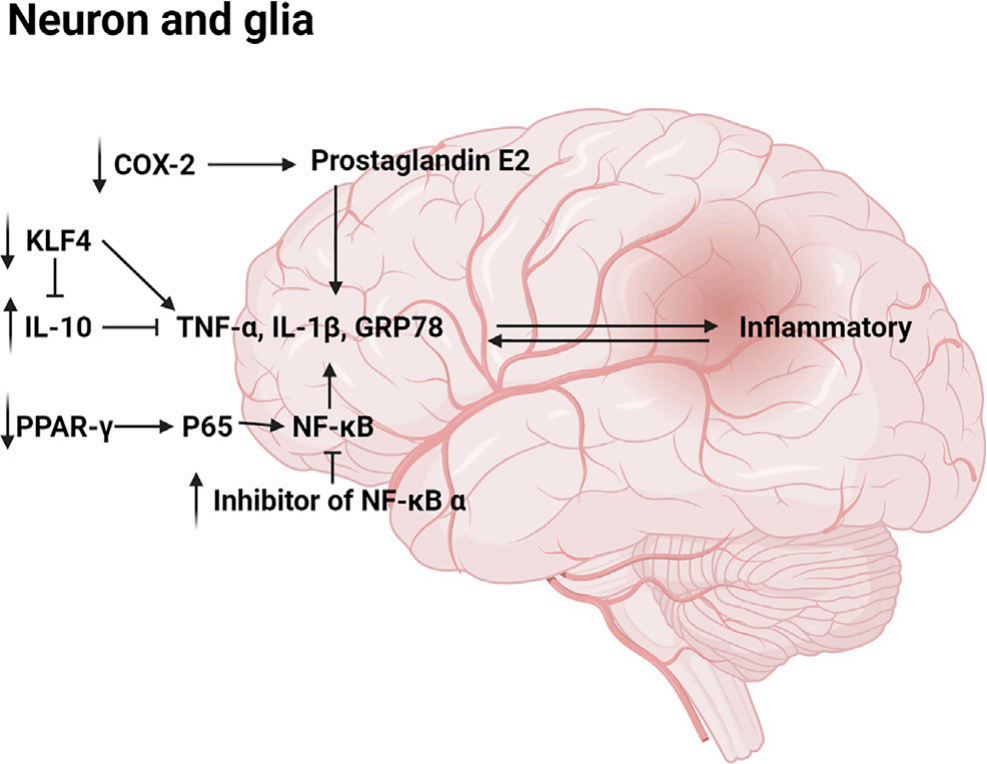
Mechanisms involved in the anti-inflammatory by neolignans.

MN **(1)** and HK **(2)** were reported to reduce IL-1β, TNF-α, GRP78, CHOP, and other proinflammatory cytokine which played a significant role in the occurrence and development of hormonal and inflammatory cascade; elevated the level of anti-inflammatory cytokine IL-10 which suppressed the synthesis of many proinflammatory mediators. (Rubio-Perez and Morillas-Ruiz, 2012; Xian et al., 2015; Zhou et al., 2019). Moreover, HK **(2)** was found to inhibit expression of COX-2 mRNA and prostaglandin E2, reducing inflammatory response (Xian et al., 2015). NF-κB pathway which could be activated by pro-inflammatory factors is considered a prototypical proinflammatory signaling pathway (Lawrence 2009; Aggarwal et al., 2012; Zhang et al., 2013). NF-κB and inhibitor of NF-κB (IκB) usually existed in an inactivated state. After being activated, NF-κB separated from IκB and entered the nucleus, promoting transcription of various inflammatory mediators such as COX-2, IL-1 β, TNF- α, IL-6, etc (Lawrence 2009). HK **(2)** was reported to downregulate NF-κB by blocking TNF-α–induced NF-κB p65 nuclear translocation and degradation of the inhibitor of NF-κB α, thereby inhibiting production of inflammatory factors (Chen et al., 2016). In another study, intravenous administration of a HK **(2)** derivative to ischemia-reperfusion rats reduced the expression level of the nuclear NF-κB p65 subunit (Bu et al., 2014). Besides, MN **(1)** could inhibit NF-κB activation and IκB degradation in RAW264.7 cells stimulated with lipopolysaccharide (Fu et al., 2013). In addition, Male TgCRND8 mice were orally administered with MN **(1)** daily for 4 consecutive months, then researchers found that the regulation of NF-κB might be related to activation of PPAR-γ (Aggarwal et al., 2012; Oboudiyat et al., 2013; Xian et al., 2020; Xie et al., 2020). Specifically, the DNA-binding activity of NF-κB was inhibited by PPAR-γ activation. Meanwhile, PPAR-γ activation induced the degradation of NF-κB p65 (Hou et al., 2012; Zhao et al., 2015). Furthermore, HK **(2)** was reported to inhibit the expression of zinc finger transcription factor Krüppel-like factor 4 (KLF4) in microglia and astrocytes, which could also regulate proinflammatory cytokine and anti-inflammatory cytokine (Rickert et al., 2018). The main pathway diagram is shown in Figure 5.

#### 4.1.5 Anti-Nerve Oxidation

**FIGURE 6:**
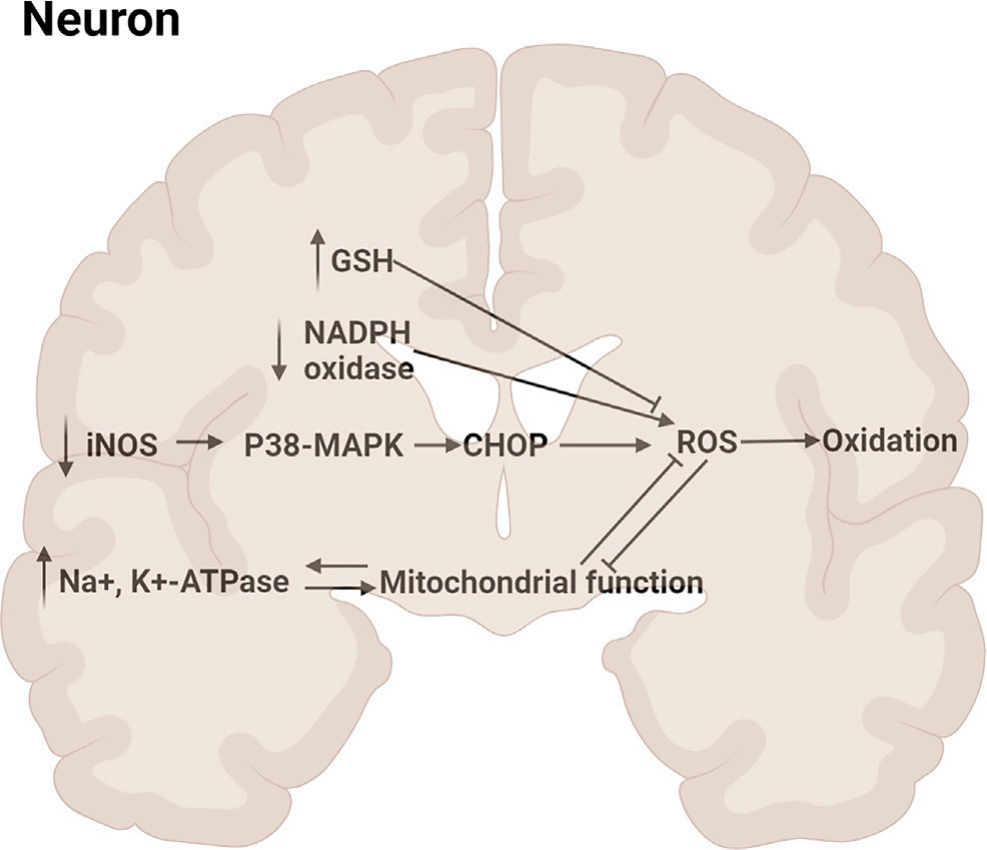
Mechanisms involved in the anti-oxidation by neolignans.

Brain was rich in oxidizable lipid which oxidative damage caused the loss of nerve cells irreversibly, leading to nerve cells dysfunction (Loo et al., 1993). It has been reported that MN **(1)** and HK **(2)** inhibited reactive oxygen species (ROS) against oxidation by inhibiting NADPH oxidase activation and Glutathione (GSH) depletion. Scop-induced mouse model with MN **(1)** treatment was found that the activities of superoxide dismutase (SOD), total nitric oxide synthase (total NOS) returned to normal. (Hoi et al., 2010; Dong et al., 2013; Chen et al., 2018). In cerebral ischemia and stroke experiments, MN **(1)** has been proved to have anti-oxidation effect which was related to iNOS/p38/MAPK pathway (Chang et al., 2003; Chang et al., 2007; Chen et al., 2014; Huang et al., 2018). Concretely, MN **(1)** downregulated p38/MAPK pathway through inhibiting inducible NO synthase (iNOS) to prevent C/EBP homologous protein (CHOP) expression, thereby reduceing the production of ROS and oxidative stress (Chen et al., 2014). Mitochondria could produce ROS, which destroyed mitochondrial intima and ATP production. The oxidative disruption of mitochondria transition pore induced leakage of mitochondrial membrane, mitochondrial swelling, ATP deletion, thereby resulting in a vicious cycling in producing ROS burst (Corona and Duchen, 2015). The production of ATP is critical for the maintenance of the activity of Na^+^, K^+^-ATPase that is inhibited sensitively by increasing of ROS (Chaturvedi and Beal, 2008). The decrease of Na^+^, K^+^-ATPase activity in the cerebral ischemia was reversed by HK **(2)**, which suggested that HK **(2)** inhibited ROS by improving synaptosomal mitochondrial metabolic function (Chen et al., 2007). The main pathway diagram is shown in Figure 6.

### 4.2 Protection of Brain Microvascular Endothelial Cells

**FIGURE 7:**
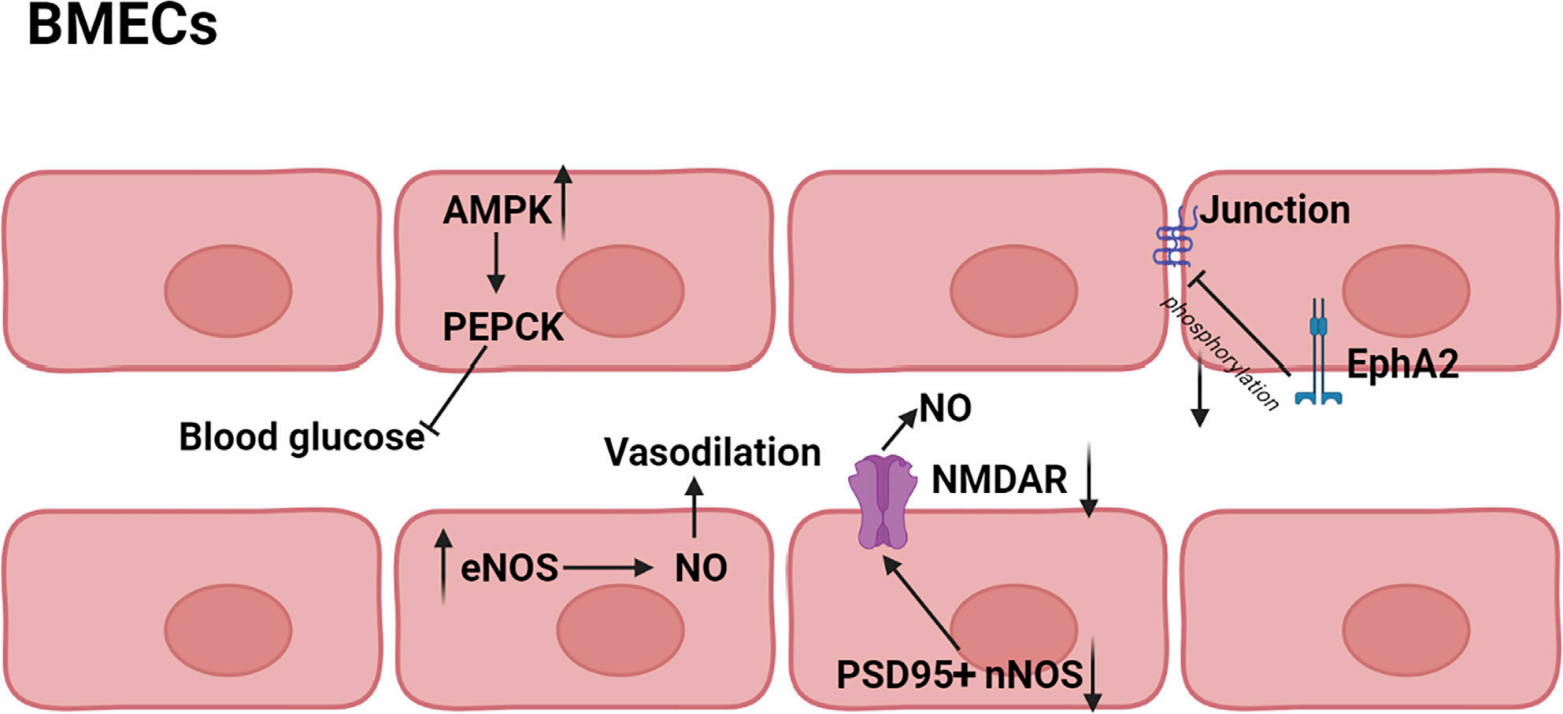
Mechanisms involved in the protection of BMECs by neolignans.

It has been shown that iNOS and neuronal NO synthase (nNOS) could produce a high level of NO, thereby inducing cell injury or death, but NO produced by endothelial NO synthase (eNOS) protected brain against stroke (Jiang et al., 2002; Li et al., 2014). HK **(2)** was reported to have effect on increasing cerebral blood supply in rats, and the mechanism might be associated with the vasodilation produced by eNOS activation and the protection of BMECs (Xiaoyan 2016). The activation of nNOS depended on translocation from cytoplasm to membrane and connection to N-methyl-D-aspartic acid receptor (NMDAR) via the scaffolding protein postsynaptic density 95 (PSD95), thereby stimulating NO production during ischemia (Fan et al., 2010; Zhou et al., 2010). HK **(2)** could block the PSD95 nNOS interaction and inhibit the translocation of nNOS from cytoplasm to membrane. In addition, HK **(2)** also inhibited NMDAR to partly (Hu et al., 2013). Adenosine 5′-monophosphate-activated protein kinase (AMPK), a serine/threonine kinase, was known to inhibit gluconeogenesis via suppression of phosphoenolpyruvate carboxy kinase (PEPCK) and other key enzymes in liver. AMPK was found to be increased by HK **(2)**, resulting in decrease of blood glucose levels (Ronnett et al., 2009). Therefore, HK **(2)** might normalize blood glucose and decrease the extent of neuronal dysfunction by preventing post-ischemic glucose intolerance (Harada et al., 2012). Interestingly, in addition to protecting individual cells, neolignans also had effect on the structure of cells. Erythropoietin-producing hepatocellular A2 (EphA2) was one of transmembrane receptors tyrosine kinases and its phosphorylation led to the destruction of the tight junction between BMECs (Zhou et al., 2010). MN **(1)** was found to inhibit phosphorylation of EphA2 and enhance formation of the tight junction between BMEC, leading to improvement of BBB (Liu et al., 2017). The main pathway diagram is shown in Figure 7.

### 4.3 Others

Prolonged and repeated stress exposure caused hyperactivation of hypothalamic-pituitary-adrenal axis (HPA), which increased the level of corticosterone and affected the cognitive function, leading to depression and anxiety (Jangra et al., 2016). It has been shown that neolignans could restore the normal active level of HPA axis, which might be related to the decrease of glucocorticoid negative feedback and increase of BDNF. HK **(2)** was reported to increase the expression of glucocorticoid receptor α, reducing negative feedback, thereafter hyperactivity of HPA axis was regulated (Wang et al., 2018). In addition, it has been proved that the increase of BDNF had significance of maintaining the normal function of HPA axis (Xu et al., 2008). These results suggested that anti-depressant effect of neolignans was the result of the synergy of BDNF and HPA axis.

## 5 Safety

In a study, models were given 625, 1250, or 2500 mg/kg extract containing 94% MN **(1)** and 1.5% HK **(2)** orally for 14 days, and researchers did not find death or clinical toxicity (Li et al., 2007). Besides, *Magnolia officinalis* extract containing high content of MN **(1)** and HK **(2)** did not been found any mutagenicity and genotoxicity, even showed partly anti-mutagenic activity *in vivo* (Bai et al., 2003; Zhang et al., 2008; Fried and Arbiser, 2009; Lee et al., 2011). It has been reported that when administered orally or intraperitoneally, the most neolignans were excreted in feces and urine within 12 h (Hattori et al., 1986). Although neolignans could cause enterohepatic circulation, researchers have not found any special hepatotoxicity (Lee et al., 2011). It has been estimated that the safe dose of MN **(1)** available for teenage is up to 1.64 mg/kg per day (Zhang et al., 2019). Besides, to date, no serious side effects on human intake of prescriptions containing MN **(1)** or HK **(2)** have been reported. In general, therapeutic doses of neolignans could be considered safe.

## 6 Conclusion

Neolignans protect the nervous system from brain diseases including AD, PD, stroke, anxiety and depression mainly by protecting nerve cells and BMECs. It has the following effects on nerve cells: 1) regulating neuronal function mainly by normalizing neurotransmitters and their receptors 2) reducing neurotoxicity through reducing APP, γ-secretase, BACE1 and stabilizing αS native conformations 3) suppressing neuronal apoptosis through reducing the levels of Bax, caspase-3 and other apoptosis-related proteins 4) anti-inflammation in nerve by PPAR-γ/NF-κB pathway 5) anti-oxidation in nerve through MAPK pathway and PI3K/Akt pathway including p38/MAPK and MAPK/ERK. The combined action of these pathways allows neolignans to have pleiotropic neuroprotective effects in different links. For BMECs, neolignans can increase blood supply, normalize blood glucose and close the connection between BMEC. In addition, neolignans also regulate the humoral environment by modulating the function of HPA axis, keeping the nervous system away from stress.

However, there are few reports on the neuroprotective effect of neolignans except MN **(1)** and HK **(2)**. The overall pharmacological effect and mechanism of neolignans can only be inferred from the existing researches of MN **(1)** and HK **(2)**. Furthermore, the clinical applications of neolignans are limited due to low bioavailability and quick metabolism, which should be paid attention to and improved. In summary, neolignans can be considered as a promising neuroprotective resource for the treatment of brain diseases, and more clinical studies are needed.

## Data Availability

The original contributions presented in the study are included in the article/Supplementary Material, further inquiries can be directed to the corresponding authors.
