# Supplementary material for: Neuroprotective Potency of Neolignans in Magnolia officinalis Cortex Against Brain Disorders
Source: Front Pharmacol. 2022 Jun 16;13:857449. doi: 10.3389/fphar.2022.857449 (PMC9244706; doi:10.3389/fphar.2022.857449)
Supplement: Supplementary file 1 [file Datasheet2.PDF]

Table 2. Effects of Neolignans and Bioactive Compounds in Parkinson's Disease

Brain Pathology	Experimental Model	Compound	Dosage	Mechanism of Action	Effect	Reference
Parkinson's Disease	Male C57BL/6 N mice, MPTP 20 mg/kg every 2 h, four times or 40 mg/kg, once	Magnolol	30 $mg.kg^{-1}.day^{-1}$, 5 days	(+) DAT, TH<br>(-) ROS	(+) Dopamine neurons<br>(-) Oxidative stress	(Muroyama et al. 2012)
	Sprague-Dawley rats, stereotaxically injected Lactacystin	Magnolol	5 weeks	(+) TH<br>(-) Ubiquitin, αS	(+) Dopamine neurons	(Li et al. 2012b)
	NMRI male mice, 6-OHDA (15 μg/3 μl)	Honokiol	0.1–5 mg/kg, Day 7 to Day 21	(+) DAT, HT, VMAT2, PPARγ<br>(-) NADPH-oxidase	(-) Oxidative stress	(Chen et al. 2018)
	αSA53T	Honokiol	100 μmol	(-) αS	(+) Dopamine neurons	(Das et al. 2018)

The symbol (+) indicates increasing. Thesymbol (-) indicates decreasing. The symbol - indicates not mentioned
