# Supplementary material for: Neuroprotective Potency of Neolignans in Magnolia officinalis Cortex Against Brain Disorders
Source: Front Pharmacol. 2022 Jun 16;13:857449. doi: 10.3389/fphar.2022.857449 (PMC9244706; doi:10.3389/fphar.2022.857449)
Supplement: Supplementary file 2 [file Datasheet4.PDF]

Table 4. Effects of Neolignans and Bioactive Compounds in Anxiety and Depression

Brain Pathology	Experimental Model	Compound	Dosage	Mechanism of Action	Effect	Reference
Anxiety and depression	Brain slices, prepared from isoflurane-anesthetized rats	Magnolol	-	(+) $GABA_A$ receptor	(+) GABAergic neurotransmission	(Alexeev et al. 2012)
	Male ddY mice, OBX surgery	Magnolol	50, 100 $mg \cdot kg^{-1}.day^{-1}$, 14 days	(+) Akt, BDNF	(+) Neurotrophic	(Matsui et al. 2016)
	Male SD rats, CUMS, 4 weeks	Magnolol	20, 40 mg/kg, 3 weeks	(+) BDNF	(+) Neurotrophic, serotonergic system	(Li et al. 2012a)
	Male SD rats, CUMS, 5 weeks	Magnolol	20, 40 mg/kg, 3 weeks	(+) GFAP	(+) Glial cells	(Li et al. 2013a)
	Male SD rats, CUMS, 6 weeks	Magnolol	3, 10, 20, 30g/kg, 4 weeks	(+) HTR1A	(+) Serotonergic system	(Xia et al. 2019)
	Male ICR mice, CORT 20 mg/kg, 21days	Magnolol	Pretreatment, 50, 100 mg/kg, 21days	(+) BDNF, 5-HT	(+) Neurotrophic, serotonergic system	(Bai et al. 2018)
	Male C57BL/6 J mice SD rats, flumazenil	Honokiol	5, 10, 20 $mg \cdot kg^{-1}$	(+) $GABA_A$ receptor	(+) GABAergic neurotransmission	(Qu et al. 2012)
	Male ICR mice	Honokiol	1 $mg.kg^{-1}.day^{-1}$, 7 days	(+) GAD65, GABA	(+) GABAergic neurotransmission	(Ku et al. 2011)
	Brain slices, prepared from isoflurane-anesthetized rats	Honokiol	-	(+) $GABA_A$ receptor	(+) GABAergic neurotransmission	(Alexeev et al. 2012)
	Male Wistar rats, CUMS, 6 weeks	Honokiol	2, 4, 8 mg/kg, 21days	(+) GRα, BDNF<br>(-) CRH, ACTH, CORT	(+) Neurotrophic, function of the HPA	(Wang et al. 2018)

					axis	
	Male Swiss albino mice, CORT 40 mg/kg 21 days	Honokiol	Pretreatment, 20 mg/kg, 21days	(+) BDNF	(+) Neurotrophic	(Pitta et al. 2013)
	Male ICR mice, FST or TST or CUMS	Honokiol, magnolol	20 mg/kg, 40 mg/kg, 1 week	(+) cAMP, BDNF, AC	(+) Serotonergic system, function of the HPA axis	(Xu et al. 2008)
	Male Swiss albino mice, restraint stress, 28 days	Honokiol	3, 10 mg/kg, last 7 days	(-) GRP78, CHOP	(-) Inflammation	(Jangra et al. 2016)

The symbol (+) indicates increasing. Thesymbol (-) indicates decreasing. The symbol - indicates not mentioned
