# Supplementary material for: Neuroprotective Potency of Neolignans in Magnolia officinalis Cortex Against Brain Disorders
Source: Front Pharmacol. 2022 Jun 16;13:857449. doi: 10.3389/fphar.2022.857449 (PMC9244706; doi:10.3389/fphar.2022.857449)
Supplement: Supplementary file 3 [file Datasheet3.PDF]

Table 3. Effects of Neolignans and Bioactive Compounds in Stroke

Brain Pathology	Experimental Model	Compound	Dosage	Mechanism of Action	Effect	Reference
Stroke	Middle cerebral artery occlusion reperfusion models of male SD rat	Magnolol	Pre-conditioning, 75 mg.kg^{-1}.day^{-1}, 7day	(+) BDNF<br>(-) Bax	(-) Apoptosis	(Liu et al. 2018)
	Organotypic hippocampal slice neonatal SD rat with 95% N_2 and 5% CO_2	Magnolol	Pre-treatment 0.01-1 mg/kg, post-treatment 1mg/kg,	(-) IL-6, TNF-α, NO	(-) Oxidative stress, inflammation<br>(+) synaptic	(Huang et al. 2018)
	SD rat, urethane,1.4 g/kg	Magnolol	20 or 40 mg/kg	(-) Free radical	(-) Oxidative stress	(Chang et al. 2003)
	Male SD rat with intracerebral haemorrhage	Magnolol	30 mg/kg	(-) IL-1β、TNF-α, MMP-9	(-) Inflammation	(Zhou et al. 2019)
	Middle cerebral artery occlusion model of stroke in rats	Magnolol	25 mg/kg, twice	(+) Bcl-2, SIRT1<br>(-) Bax, ac-FOXO1, IL-1β, TNF-α	(-) Apoptosis, inflammation	(Kou et al. 2017)
	Male SD rat, bilateral carotid arteries to be occluded ligated with 4-0 nylon	Magnolol	10 mg/kg, 30 mg/kg	(-) Nitrotyrosine, 4-HNE, iNOS, CHOP, p-p38/MAPK<br>(+) p-Akt, NF-κB	(-) Oxidative stress	(Chen et al. 2014)
	SD rats, block bilateral carotid arteries, 30 min, re-perfusing, 24 h, collect brains	Honokiol	0.7~70g/kg	(-) NF-κB, TNF-α, intracellular NO, RANTES/CCL5	(-) Inflammation	(Zhang et al. 2013)
	ECs	Honokiol	-	(+) IκBα	(-) Inflammation	(Chen et al. 2016)

	Male mice, subjected to middle cerebral artery occlusion for 45 min	Honokiol	10 μg/kg	(-) NF-κB<br>(+) Na^+, K^+-ATPase activities	(-) Oxidative stress<br>(+) Mitochondrial function	(Chen et al. 2007)

The symbol (+) indicates increasing. Thesymbol (-) indicates decreasing. The symbol - indicates not mentioned
