# Supplementary material for: Neuroprotective Potency of Neolignans in Magnolia officinalis Cortex Against Brain Disorders
Source: Front Pharmacol. 2022 Jun 16;13:857449. doi: 10.3389/fphar.2022.857449 (PMC9244706; doi:10.3389/fphar.2022.857449)
Supplement: Supplementary file 4 [file Datasheet1.PDF]

Table 1. Effects of Neolignans and Bioactive Compounds in Alzheimer's disease

Brain Pathology	Experimental Model	Compound	Dosage	Mechanism of Action	Effect	Reference
Alzheimer's disease	BV2 cells, FAM-tagged Aβ (1-42) for 4 h	Magnolol	Pre-conditioning, 10 μM, 18 h	(+) PPAR-γ<br>(-) ROS	(-) Aβ, oxidative stress	(Xie et al. 2020)
	TgCRND8 Mice	Magnolol	20 $mg.kg^{-1}.day^{-1}$, 40 $mg.kg^{-1}.day^{-1}$, 4 months	(+) P-GSK-3β(Ser9)/GSK-3β, p-Akt (Ser473)/Akt, p-NF-κB p65/NF-κB p65	(-) Inflammation, Aβ<br>(+) Synaptic	(Xian et al. 2020)
	Day 3 to Day 8, Scopolamine (2 $mg.kg^{-1}$)-induced mice	Magnolol	Day 1 to Day 8, 15 $mg.kg^{-1}.day^{-1}$, 25 $mg.kg^{-1}.day^{-1}$, 35 $mg.kg^{-1}.day^{-1}$	(+) TNOS, SOD, AChE<br>(-) MDA	(+) NO, ACh	(Li et al. 2013b)
	PC12 cells grown in DMEM containing 50 ng/mL NGF with Aβ	Magnolol	-	(-) ROS, intracellular calcium elevation, caspase-3 activity	(-) Aβ, oxidative stress	(Hoi et al. 2010b)
	Male SAMP8/TaSlc and SAMR1/TaSlc mice	Magnolol	1,10 $mg.kg^{-1}.day^{-1}$, 14 days	(+) p-Akt, cholinergic neuron<br>(-) Number of ChAT-positive cells	(+) Neurotrophic	(Matsui et al. 2009)
	SH-SY5Y cells exposed to acrolein (1, 5, 10, or 20 μM), 24 h	Magnolol	8、16、32μM, 2 h	JNK/mitochondria/caspase, PI3K/MEK/ERK, PI3K/Akt/FoxO1 signaling pathways	(-) Oxidative stress<br>(+) Cell survival	(Dong et al. 2013)
	PC12 cells grown in DMEM containing 50 ng/mL NGF with Aβ	Honokiol	-	(-) ROS, intracellular calcium elevation, caspase-3 activity	(-) Aβ, Oxidative stress	(Hoi et al. 2010a)
	Male SAMP8/TaSlc and	Honokiol	0.1, 1 $mg.kg^{-1}.day^{-1}$, 14	(+) P-Akt, cholinergic neuron	(+) Neurotrophic	(Matsui et al. 2009)

	SAMR1/TaSlc mice		days	(-) Number of ChAT-positive cells		
	Adult Swiss albino mice	Honokiol	3，10 mg.kg^{-1}, 7 days	(-) Proinflammatory cytokines, endoplasmic reticulum stress markers	(-) Inflammation, endoplasmic reticulum stress	(Jangra et al. 2016)

The symbol (+) indicates increasing. Thesymbol (-) indicates decreasing. The symbol - indicates not mentioned
